# A comparison of myopia control in European children and adolescents with defocus incorporated multiple segments (DIMS) spectacles, atropine, and combined DIMS/atropine

**DOI:** 10.1371/journal.pone.0281816

**Published:** 2023-02-16

**Authors:** Paolo Nucci, Andrea Lembo, Irene Schiavetti, Rakhee Shah, David Francis Edgar, Bruce John William Evans

**Affiliations:** 1 Department of Biomedical, Surgical and Dental Sciences, University of Milan, Milan, Italy; 2 Department of Biomedical, Surgical and Dental Sciences, University of Milan San Giuseppe Hospital, Milan, Italy; 3 Department of Health Sciences, Section of Biostatistics, University of Genoa, Genoa, Italy; 4 Research Department, Institute of Optometry, London, United Kingdom; 5 Department of Optometry and Visual Sciences, School of Health and Psychological Sciences, University of London, London, United Kingdom; National Eye Institute, UNITED STATES

## Abstract

**Purpose:**

To evaluate the efficacy of a myopia control spectacle lens (DIMS) at slowing the progression of myopia in a population of European children in comparison with 0.01% atropine and combined DIMS and atropine.

**Methods:**

The study was a non-randomised experimenter-masked prospective controlled observational study of individuals aged 6–18 years with progressing myopia but no ocular pathology. Participants were allocated, according to patient/parent choice, to receive 0.01% atropine eyedrops, DIMS (Hoya^®^ MiyoSmart^®^) spectacles, combined atropine+DIMS or single vision spectacle lenses (control group). The key outcome variables, cycloplegic autorefraction spherical equivalent refraction (SER) and axial length (AL), were measured at baseline and after three, six, and 12 months.

**Results:**

Of the 146 participants (mean age 10.3y ±3.2), 53 received atropine, 30 DIMS spectacles, 31 atropine+DIMS, and 32 single vision control spectacles. Generalized linear mixed model analysis revealed for SER, whilst controlling for age and SER at baseline, at each stage all treatment groups had significantly reduced progression compared with the control group (p<0.016). For AL, whilst controlling for baseline age and AL, at 6 and 12 months all treatment groups had significantly less progression than the control group (p<0.005). For SER only, in pairwise comparisons at 12 months the atropine+DIMS group had significantly reduced progression compared with the DIMS only and Atropine only groups (p<0.001).

**Conclusion:**

In a European population, DIMS and atropine are effective at reducing myopia progression and axial elongation in progressing myopia and are most successful at reducing myopia progression when used in combination.

## Introduction

Approximately 30% of Europeans are myopic [1]. The prevalence of myopia is increasing worldwide and it is estimated that in 2050, 50% of the world population will be myopic [2]. Many factors are recognized, both genetic and environmental, that influence the development and progression of myopia, such as the education level and sunlight exposure [3, 4]. Myopia, especially high myopia, is associated with an increased risk of sight-threatening eye disease [5, 6], creating a long-term burden on public health [2, 7] and economies [8].

There is growing interest in methods that slow the progression of myopia [9], including atropine eye drops [10], dual focus contact lenses and spectacle lenses, and orthokeratology [11, 12]. Atropine has been widely used effectively for myopia control [13].

Defocus incorporated multiple segments (DIMS) spectacle lenses are designed to slow myopia progression in children, based on the principle of peripheral myopic defocus and simultaneous vision. They are a dual focus spectacle lens consisting of a central optical zone for correcting distance refractive error, and an annulus comprising several hundred circular segments, each ~1 mm in diameter with a relative positive power of 3.50D equally distributed throughout the mid-peripheral area in a honeycomb pattern [14]. DIMS spectacles reduce the progression of myopia and reduce axial elongation by 50–60% compared to single vision (SV) lenses [14–16]. The optical properties of DIMS spectacles [17] cause minimal [18] or no [16] adverse effects on vision.

The literature reveals no trials of DIMS in European populations and no studies comparing atropine with DIMS. It is believed that different mechanisms underly the benefit from atropine (non-accommodative, possibly via acting directly on receptors in the sclera) and optical approaches such as DIMS (reducing relative hyperopic defocus) [19]. Therefore, it is hypothesised that their combined use may create an additive effect, which to date has not been explored.

The goals of this study are to evaluate the efficacy of DIMS in slowing the progression of myopia in a population of European children in comparison with atropine and combined DIMS and atropine.

## Materials and methods

### Study design

The study was a prospective controlled observational study carried out in a paediatric ophthalmology clinic setting. The clinic has a reputation for myopia control and participants were highly motivated to pursue treatment, but often attended the clinic because of a preference for a specific intervention and therefore random allocation to study groups was not possible. Measurements of visual acuity (VA), cycloplegic autorefraction spherical equivalent refraction (SER), and axial length (AL) were taken by masked observers following a fixed protocol.

Potential participants underwent a full ophthalmological assessment including symptoms and history, presenting VA with pre-study spectacles, orthoptic testing, refraction (including cycloplegic autorefraction), and dilated fundoscopy. Suitable participants (see below) were provided with information on three options for myopia control: 0.01% atropine, DIMS spectacles, or combined 0.01% atropine+DIMS. These options were discussed with patients, parents, and clinicians (PN, AL) and participants and their parents were free to choose their preferred option, or to continue in single vision spectacles. Some families were hesitant to undertake a long-term pharmacological or novel optical treatment and therefore opted not to undertake myopia control at that time and instead to join the control group and wear single vision spectacles.

For all participants, written informed consent was obtained from parents/guardians. Participants were provided with their interventions and follow-up arranged after 3, 6, and 12 months. The outcome variables were assessed at each follow-up. The importance of attendance at follow-up was stressed to all participants, and telephone reminders were used together with rebooking of missed appointments to encourage attendance.

The study received approval from the Ethics Committee of the University of Milan and was performed in accordance with the ethical standards as laid down in the 1964 Declaration of Helsinki and its later amendments.

### Participants

The selection criteria are in Table 1. The target sample size was at least 30 participants in each group. Myopia milder than -0.50D (SER) was excluded to avoid potential difficulties persuading participants with minimal myopia to wear spectacles. Myopia higher than 4.00D was excluded to decrease the risk of any participants having syndromic myopia.

**Table 1:** Selection criteria.

Inclusion criteria	• Children/adolescents aged 6–18 years • Italian/European ethnicity • Myopia with SER from -0.50D to -4.00D • Astigmatism not more than 2.50DC • Anisometropia under 1.25D
Exclusion criteria	• Genetic syndromes suspected (e.g., Stickler, Marfan etc.) • Other eye diseases (such as glaucoma, juvenile cataracts or retinal abnormalities, any form of strabismus) • Myopia progression in the last year of less than 0.50D SER in either eye

### Interventions

DIMS spectacles (Hoya^®^ MiyoSmart^®^) were prescribed and dispensed according to the manufacturer’s fitting guide, with participants instructed to wear the spectacles as close to all waking hours as practical (e.g., not for bathing or swimming). For those receiving atropine, 0.01% drops (ATOM galenic formulation) [20] were used, with one drop being instilled in each eye every night, before sleeping. All participants throughout the study were free to ask for a re-evaluation of their myopic prescription.

### Outcome variables

The outcome variables were always assessed in the same room with lighting set at 600 Lux by the same team of four orthoptists, all of whom were masked to the participants’ interventions.

The primary outcome variables were the change in SER and in AL. Cycloplegic autorefraction was carried out after instillation of cyclopentolate (Allergan Ciclolux^®^ 10mg/ml), with two drops in each eye instilled five minutes apart and refraction (Retinomax^®^) after 30 minutes (set to 0.25D, median of 3 readings for each measurement). AL was measured in each eye with a Zeiss IOLMaster^®^ instrument.

To preserve masking, at each follow-up, VA testing was repeated with the refractive error determined at baseline worn in an optometric trial frame. An ETDRS LogMAR [21] chart was used in a computerised system that presented random letters. The clinical procedure was to use whole line scoring (criterion: 3/5 letters correctly read) in decimal units, with a test ceiling of 1.0 (0.0 LogMAR) acuity. The limitations for this secondary variable are considered further in the Discussion.

### Statistical analysis

Continuous variables were summarized as mean with standard deviation and median with interquartile range. Categorical data were expressed with frequency and percentage. Differences across the groups in baseline characteristics were evaluated by the Kruskal-Wallis test.

A generalized linear mixed model (GLMM) was applied to evaluate the treatment effect on SER, AL and VA. The model included treatment and the interaction time by treatment as fixed effect, age and baseline value as fixed covariate; and subject and eye (right or left) as random effect. Multiple comparisons were adjusted using sequential Bonferroni. Two-sided p-values of less than 0.05 were considered statistically significant. IBM SPSS Statistics V.24.0 (IBM Corp. Released 2016, Armonk, New York, USA: IBM Corp), was used for statistical analysis.

## Results

### Study population

One hundred and forty-six participants with myopia and a mean age of 10.3 (± 3.21) years were enrolled and allocated to the four groups: DIMS (N = 30), atropine (N = 53), atropine+DIMS (N = 31), and single vision control (N = 32). Baseline characteristics are in Table 2. Since participants were not randomly allocated to groups, the groups differed significantly in some characteristics at baseline. Specifically, pairwise comparisons revealed the following statistically significant (p<0.05) differences: the DIMS group was older than other groups, atropine group was younger than the other groups; the control group and atropine group had lower values of SER than atropine+DIMS, and lower values than the DIMS group; and the atropine+DIMS group had higher values of AL compared to the control group and compared to the atropine group. However, the GLMM analyses were corrected for differences in these factors at baseline.

**Table 2:** Participant characteristics at baseline^1^.

	Total (N = 146)	Control (N = 32)	DIMS (N = 30)	Atropine (N = 53)	Atropine+DIMS (N = 31)	p
**Age in years**	10.28 ± 3.21	11.34 ± 3.96	13.37 ± 2.22	8.17 ± 1.84	9.81 ± 2.06	<0.001
	10 (7 to 13)	11 (8 to 15.5)	14 (12 to 15)	8 (7 to 9)	10 (8 to 11)	
**Baseline SER (D), right**	-1.77 ± 0.70	-1.54 ± 0.74	-1.97 ± 0.69	-1.56 ± 0.69	-2.16 ± 0.46	<0.001
	-2.00 (-2.25 to -1.25)	-1.62 (-2.00 to -0.87)	-2.00 (-2.25 to -1.75)	-1.75 (-2.00 to -1.00)	-2.00 (-2.25 to -1.75)	
**Baseline SER (D), left**	-1.77 ± 0.70	-1.54 ± 0.74	-1.97 ± 0.69	-1.56 ± 0.69	-2.16 ± 0.46	<0.001
	-2.00 (-2.25 to -1.25)	-1.62 (-2.00 to -0.87)	-2.00 (-2.25 to -1.75)	-1.75 (-2.00 to -1.00)	-2.00 (-2.25 to -1.75)	
**Baseline AL (mm), right**	24.79 ± 0.80	24.64 ± 0.79	24.87 ± 0.71	24.61 ± 0.87	25.16 ± 0.64	0.029
	25.01 (24.09 to 25.46)	24.46 (24.08 to 25.44)	24.91 (24.12–25.52)	24.33 (24.01–25.33)	25.12 (24.95 to 25.61)	
**Baseline AL (mm), left**	24.80 ± 0.80	24.64 ± 0.79	24.83 ± 0.71	24.66 ± 0.89	25.16 ± 0.63	0.05
	25.01 (24.11 to 2.52)	24.46 (24.08 to 25.44)	24.76 (24.11–25.52)	24.71 (23.89–25.33)	25.12 (24.95 to 25.56)	

^1^Results are expressed as mean and standard deviation (first line) and median and inter-quartile range (second line)

Limitations in the way visual acuity was assessed (whole line scoring and a test ceiling of 1.0 decimal) mean that at baseline, the mean, median, and limits of inter-quartile range of all groups were each 1.0 decimal. None of the participants had prior experience of myopia control.

All participants attended all three follow-up visits. Some appointments had to be rescheduled when participants failed to attend, but all appointments took place within 4 weeks of the due date. No adverse events were reported.

### Primary outcomes: SER & AL

**Fig 1:**
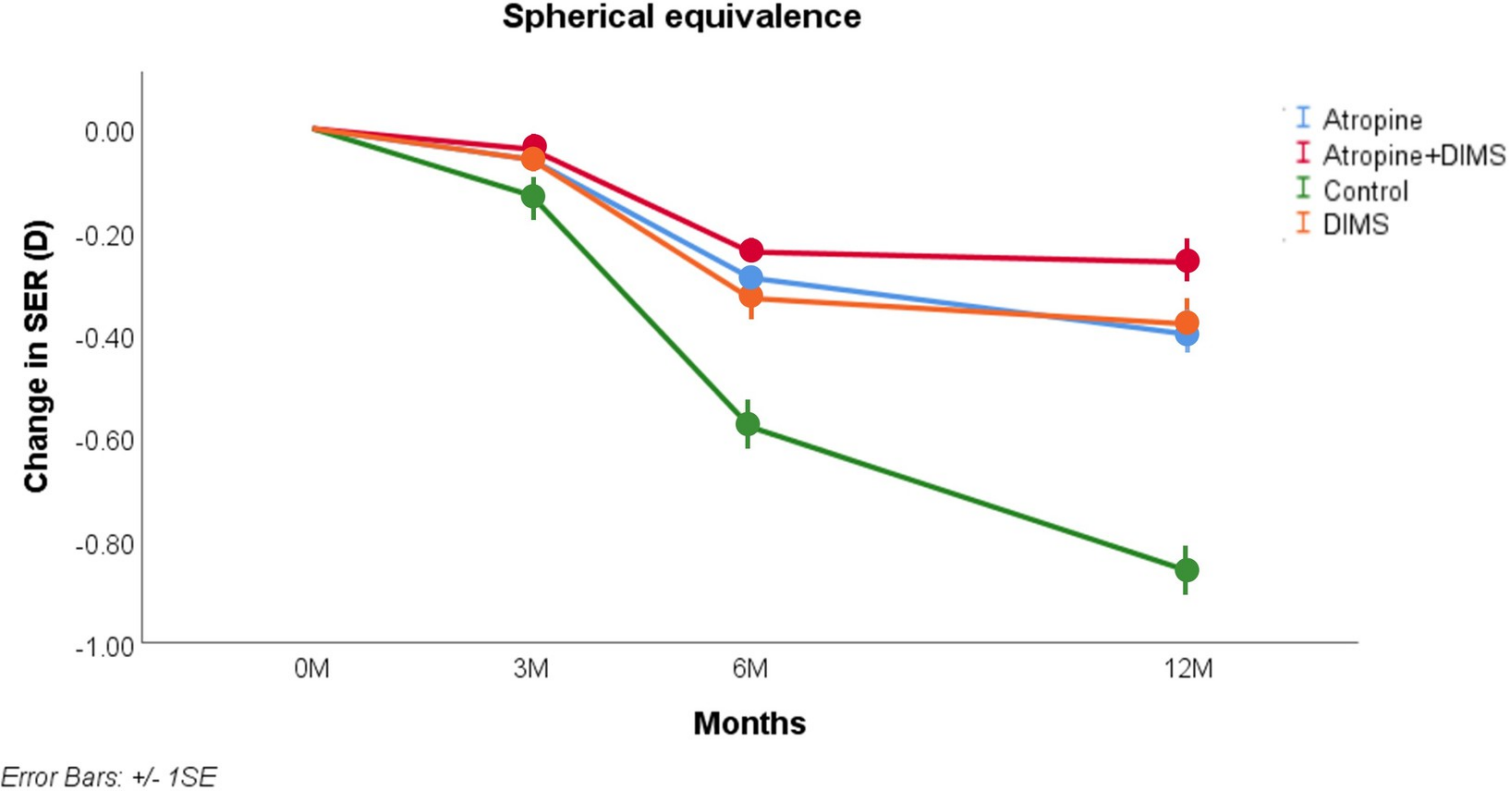
Model-adjusted mean and SE of myopia progression (SER) from baseline to 12 months.

**Fig 2:**
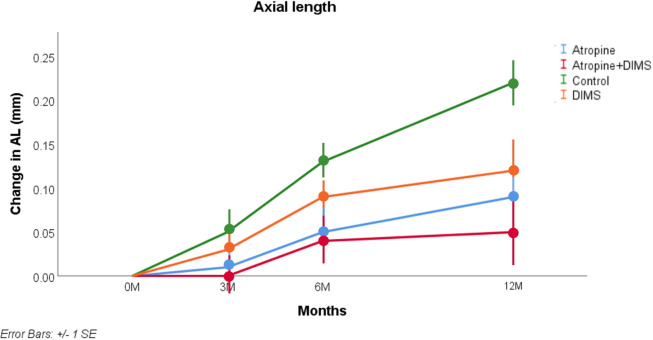
Model-adjusted mean and SE of change in axial length from baseline to 12 months.

The results are illustrated in Figs 1 and 2 and the statistical analysis is summarised in Tables 3 and 4. For SER at 12 months (Fig 1 and Table 3), controlling for age and SER at baseline, the key interaction (comparison with control group) was statistically significant (p<0.001) for all three treatment groups.

**Table 3:** Treatment effect over time on SER.

Months	Control	Atropine	Atropine + DIMS	DIMS	P value
3M	-1.916 (0.017) (-1.949 to -1.882)	-1.821 (0.014) (-1.848 to -1.794)	-1.772 (0.017) (-1.806 to -1.738)	-1.845 (0.019) (-1.882 to -1.808)	Overall: p<0.001
					Control vs Atropine: <0.001
					Control vs Atropine + DIMS: <0.001
					Control vs DIMS: 0.015
					Atropine vs Atropine + DIMS: 0.05
					Atropine vs DIMS: 0.33
					Atropine + DIMS vs DIMS: 0.017
6M	-2.362 (0.018) (-2.397 to -2.326)	-2.070 (0.015) (-2.099 to -2.042)	-1.978 (0.019) (-2.014 to -1.941)	-2.087 (0.020) (-2.126 to -2.048)	Overall: p<0.001
					Control vs Atropine: <0.001
					Control vs Atropine + DIMS: <0.001
					Control vs DIMS: <0.001
					Atropine vs Atropine + DIMS: <0.001
					Atropine vs DIMS: 0.53
					Atropine + DIMS vs DIMS: <0.001
12M	-2.641 (0.028) (-2.696 to -2.587)	-2.165 (0.022) (-2.208 to -2.122)	-2.002 (0.028) (-2.058 to -1.946)	-2.153 (0.029) (-2.210 to -2.095)	Overall: p<0.001
					Control vs Atropine: <0.001
					Control vs Atropine + DIMS: <0.001
					Control vs DIMS: <0.001
					Atropine vs Atropine + DIMS: <0.001
					Atropine vs DIMS: 0.74
					Atropine + DIMS vs DIMS: 0.001

^1^Results are expressed as mean and standard error and 95%CI

Continuous predictors are fixed at the following values: Age = 10.281, Ser at baseline = -1.7671

**Table 4:** Treatment effect over time on AL.

Months	Control	Atropine	Atropine + DIMS	DIMS	P value
3M	24.840 (0.007) (24.827 to 24.853)	24.797 (0.006) (24.786to 24.808)	24.802 (0.007) (24.789 to 24.815)	24.817 (0.008) (24.802 to 24.832)	Overall: p<0.001
					Control vs Atropine: <0.001
					Control vs Atropine + DIMS: <0.001
					Control vs DIMS: 0.06
					Atropine vs Atropine + DIMS: 0.56
					Atropine vs DIMS: 0.16
					Atropine + DIMS vs DIMS: 0.31
6M	24.916 (0.012) (24.892 to 24.939)	24.853 (0.009) (24.834 to 24.872)	24.843 (0.012) (24.819 to 24.867)	24.859 (0.013) (24.834 to 24.884)	Overall: p<0.001
					Control vs Atropine: <0.001
					Control vs Atropine + DIMS: <0.001
					Control vs DIMS: 0.004
					Atropine vs Atropine + DIMS: 0.99
					Atropine vs DIMS: 0.99
					Atropine + DIMS vs DIMS: 0.99
12M	25.010 (0.014) (24.982 to 25.037)	24.887 (0.011) (24.866 to 24.909)	24.851 (0.014) (24.824 to 24.879)	24.883 (0.015) (24.854 to 24.912)	Overall: p<0.001
					Control vs Atropine: <0.001
					Control vs Atropine + DIMS: <0.001
					Control vs DIMS: <0.001
					Atropine vs Atropine + DIMS: 0.13
					Atropine vs DIMS: 0.82
					Atropine + DIMS vs DIMS: 0.26

^1^Results are expressed as mean and standard error and 95%CI

Continuous predictors are fixed at the following values: Age = 10.281, Axial length at baseline = 24.7916

For AL at 12 months (Fig 2 and Table 4), controlling for age and AL at baseline, the results of each treatment group differed significantly from the control group (p<0.001). Figs 1 and 2 reveal the effects of each treatment is sustained over the year of the study, and indeed the AL appears more stable in the last six months than in the first six months. Considering Figs 1 and 2, the slowest progression occurred in the group receiving the combined atropine+DIMS intervention.

### Secondary analysis: Changes in VA

When controlling for baseline age and VA, the deterioration in VA (measured with the refractive error found at baseline) at six months and 12 months was significantly less in each treatment group than in the control group (p<0.001).

## Discussion

Although the present study is not a formal randomised controlled trial, the results are novel in reporting the effects of DIMS spectacle lenses and atropine, in isolation and combined, in a European population. The findings indicate that 0.01% atropine and DIMS are individually effective in this population, and even more effective when combined.

It has become commonplace in the literature on myopia control to use percentage reduction in progression as an index to describe treatment effect, but Brennan and colleagues cautioned that this can be misleading [22]. However, for comparison with previous literature, it is reassuring that after one year the percentage reduction in myopia progression (raw data, relative to the control group) in the atropine (57% in SER and 62% in AL) and DIMS (57% SER, 57% AL) groups is comparable to that quoted by other researchers, and is more marked in the combined atropine+DIMS group (70% SER, 77% AL).

The proportion of participants who showed, from baseline to 12 months, no increase in axial length was in the DIMS group 10%, in the atropine group 15%, and in the atropine+DIMS group 18%, compared with only 2% of the control group. Lam and colleagues reported that 14% of children wearing DIMS showed no axial elongation over 2 years [14], and Bao et al with another lenslet design found no axial elongation in 28% of participants after one year [23].

Recent studies indicate a dose effect of atropine and suggest that 0.05% may be the optimum dose for balancing efficacy with side effects [24], although an age effect is evident with younger ages benefitting from higher doses [25]. However, this research, like most on myopia control, concentrates on Asian populations. The reduced pigmentation in populations of European racial origin raises the possibility that 0.01% may be more effective, although at present evidence is lacking. Joachimsen et al report more relevant side effects of 0.05% topical atropine in young Caucasian children, potentially compromising acceptance and compliance with this dosage [26].

### Strengths and limitations

Most studies of atropine and all trials of DIMS have been on Asian populations and this study is an important extension of this work to a European population. Another strength of the study is the novel inclusion of a combined atropine+DIMS group.

A major weakness is that participants chose which intervention they received: there was no random allocation to groups. The clinic in which participants were examined had a reputation for myopia control with atropine and therefore more participants elected to be in this group. Although random allocation to groups is desirable to reduce the risk of bias, it has the disadvantage of making results less relevant to clinical practice. This is one reason why it has been argued that hierarchies of evidence should be replaced by an acceptance of the need for a diversity of approaches, including non-randomised observational studies [27].

Another limitation is that the study was single-masked, and participants were not masked to the treatment they received. In mitigation, it is helpful that the measurements of refractive error and AL were objective and taken by clinicians who were masked to the treatment that each participant was receiving. The method of measuring VA is suboptimal (whole line scoring and test ceiling of 1.0 decimal (0.0 LogMAR)).

The duration of the study of one year is similar to some other research in this field [23, 24], but does not address questions about long-term efficacy. Other research has addressed this issue [28]. Another question is about rebound effects when treatment is ceased. A rebound effect often occurs when atropine is withdrawn [28, 29], but may be avoided by tapering [30]. Many years ago it was hypothesised that optical interventions for myopia control work in a more natural way than atropine, through normalising the plane of the peripheral image shell nearer to the retina, and therefore are unlikely to cause a rebound effect on cessation of treatment [19]. Evidence from optical treatment using contact lenses supports this hypothesis [31], but this question has not yet been addressed with lenslet designs.

The study population represent individuals and families who are motivated to pursue myopia control and were attending a clinic that built a strong rapport with patients, which no doubt contributed to the high compliance rate. It is not known whether the findings will apply to less motivated populations. Similarly, it is not known whether the novel findings concerning combined atropine+DIMS apply to populations with other racial origins.

## Conclusions

In conclusion, DIMS and 0.01% atropine appear to offer efficacious interventions for slowing myopic axial elongation and combining these two treatments seems most effective at slowing myopia progression. To the best of our knowledge, this is the first study of this type to be conducted on European participants.

## References

[1] Williams KM, Verhoeven VJ, Cumberland P, Bertelsen G, Wolfram C, Buitendijk GH, et al. Prevalence of refractive error in Europe: the European Eye Epidemiology (E(3)) Consortium. Eur J Epidemiol. 2015;30(4):305–15. Epub 2015/03/18. doi: 10.1007/s10654-015-0010-0 ; PubMed Central PMCID: PMC4385146. 25784363 PMC4385146

[2] Holden BA, Fricke TR, Wilson DA, Jong M, Naidoo KS, Sankaridurg P, et al. Global Prevalence of Myopia and High Myopia and Temporal Trends from 2000 through 2050. Ophthalmology. 2016;123(5):1036–42. Epub 2016/02/11. doi: 10.1016/j.ophtha.2016.01.006 . 26875007

[3] Verhoeven VJ, Buitendijk GH, Rivadeneira F, Uitterlinden AG, Vingerling JR, Hofman A, et al. Education influences the role of genetics in myopia. Eur J Epidemiol. 2013;28(12):973–80. Epub 2013/10/19. doi: 10.1007/s10654-013-9856-1 ; PubMed Central PMCID: PMC3898347. 24142238 PMC3898347

[4] Hysi PG, Choquet H, Khawaja AP, Wojciechowski R, Tedja MS, Yin J, et al. Meta-analysis of 542,934 subjects of European ancestry identifies new genes and mechanisms predisposing to refractive error and myopia. Nat Genet. 2020;52(4):401–7. Epub 2020/03/30. doi: 10.1038/s41588-020-0599-0 ; PubMed Central PMCID: PMC7145443. 32231278 PMC7145443

[5] Saw SM, Gazzard G, Shih-Yen EC, Chua WH. Myopia and associated pathological complications. Ophthalmic Physiol Opt. 2005;25(5):381–91. doi: 10.1111/j.1475-1313.2005.00298.x . 16101943

[6] Haarman AEG, Enthoven CA, Tideman JWL, Tedja MS, Verhoeven VJM, Klaver CCW. The Complications of Myopia: A Review and Meta-Analysis. Invest Ophthalmol Vis Sci. 2020;61(4):49. Epub 2020/04/30. doi: 10.1167/iovs.61.4.49 ; PubMed Central PMCID: PMC7401976. 32347918 PMC7401976

[7] Saw SM, Gazzard G, Shih-Yen EC, Chua WH. Myopia and associated pathological complications. Ophthalmic and Physiological Optics. 2005;25(5):381–91. doi: 10.1111/j.1475-1313.2005.00298.x 16101943

[8] Fricke TR, Holden BA, Wilson DA, Schlenther G, Naidoo KS, Resnikoff S, et al. Global cost of correcting vision impairment from uncorrected refractive error. Bull World Health Organ. 2012;90(10):728–38. Epub 2012/10/31. doi: 10.2471/BLT.12.104034 ; PubMed Central PMCID: PMC3471057. 23109740 PMC3471057

[9] Vagge A, Ferro Desideri L, Nucci P, Serafino M, Giannaccare G, Traverso CE. Prevention of Progression in Myopia: A Systematic Review. Diseases. 2018;6(4). Epub 2018/10/03. doi: 10.3390/diseases6040092 ; PubMed Central PMCID: PMC6313317. 30274355 PMC6313317

[10] Sacchi M, Serafino M, Villani E, Tagliabue E, Luccarelli S, Bonsignore F, et al. Efficacy of atropine 0.01% for the treatment of childhood myopia in European patients. Acta Ophthalmol. 2019;97(8):e1136–e40. Epub 2019/06/15. doi: 10.1111/aos.14166 . 31197953

[11] Prousali E, Haidich A-B, Fontalis A, Ziakas N, Brazitikos P, Mataftsi A. Efficacy and safety of interventions to control myopia progression in children: an overview of systematic reviews and meta-analyses. BMC Ophthalmology. 2019;19(1):106. doi: 10.1186/s12886-019-1112-3 31072389 PMC6506938

[12] Wildsoet CF, Chia A, Cho P, Guggenheim JA, Polling JR, Read S, et al. IMI—Interventions Myopia Institute: Interventions for Controlling Myopia Onset and Progression Report. Invest Ophthalmol Vis Sci. 2019;60(3):M106–M31. doi: 10.1167/iovs.18-25958 . 30817829

[13] Zhao C, Cai C, Ding Q, Dai H. Efficacy and safety of atropine to control myopia progression: a systematic review and meta-analysis. BMC Ophthalmol. 2020;20(1):478. Epub 2020/12/09. doi: 10.1186/s12886-020-01746-w ; PubMed Central PMCID: PMC7720573. 33287746 PMC7720573

[14] Lam CSY, Tang WC, Tse DY, Lee RPK, Chun RKM, Hasegawa K, et al. Defocus Incorporated Multiple Segments (DIMS) spectacle lenses slow myopia progression: a 2-year randomised clinical trial. Br J Ophthalmol. 2020;104(3):363–8. Epub 2019/05/31. doi: 10.1136/bjophthalmol-2018-313739 ; PubMed Central PMCID: PMC7041503. 31142465 PMC7041503

[15] Lam CS, Tang WC, Lee PH, Zhang HY, Qi H, Hasegawa K, et al. Myopia control effect of defocus incorporated multiple segments (DIMS) spectacle lens in Chinese children: results of a 3-year follow-up study. Br J Ophthalmol. 2021. Epub 2021/03/19. doi: 10.1136/bjophthalmol-2020-317664 . 33731364 PMC9340033

[16] Lam CSY, Tang WC, Qi H, Radhakrishnan H, Hasegawa K, To CH, et al. Effect of Defocus Incorporated Multiple Segments Spectacle Lens Wear on Visual Function in Myopic Chinese Children. Transl Vis Sci Technol. 2020;9(9):11. Epub 2020/09/04. doi: 10.1167/tvst.9.9.11 ; PubMed Central PMCID: PMC7442864. 32879767 PMC7442864

[17] Jaskulski M, Singh NK, Bradley A, Kollbaum PS. Optical and imaging properties of a novel multi-segment spectacle lens designed to slow myopia progression. Ophthalmic Physiol Opt. 2020;40(5):549–56. Epub 2020/08/19. doi: 10.1111/opo.12725 . 32808381

[18] Lu Y, Lin Z, Wen L, Gao W, Pan L, Li X, et al. The Adaptation and Acceptance of Defocus Incorporated Multiple Segment Lens for Chinese Children. Am J Ophthalmol. 2020;211:207–16. Epub 2019/12/15. doi: 10.1016/j.ajo.2019.12.002 . 31837317

[19] Holden B, Sankaridurg P, Smith E, Aller T, Jong M, He M. Myopia, an underrated global challenge to vision: where the current data takes us on myopia control. Eye. 2014;28(2):142–6. doi: 10.1038/eye.2013.256 24357836 PMC3930268

[20] Chia A, Chua WH, Cheung YB, Wong WL, Lingham A, Fong A, et al. Atropine for the treatment of childhood myopia: safety and efficacy of 0.5%, 0.1%, and 0.01% doses (Atropine for the Treatment of Myopia 2). Ophthalmology. 2012;119(2):347–54. doi: 10.1016/j.ophtha.2011.07.031 21963266

[21] Ferris FL, Kassoff A, Bresnick GH, Bailey I. New visual acuities charts for clinical research. American Journal Ophthalmology. 1982;94:91–6. 7091289

[22] Brennan NA, Toubouti YM, Cheng X, Bullimore MA. Efficacy in myopia control. Prog Retin Eye Res. 2020:100923. Epub 2020/12/01. doi: 10.1016/j.preteyeres.2020.100923 . 33253901

[23] Bao J, Yang A, Huang Y, Li X, Pan Y, Ding C, et al. One-year myopia control efficacy of spectacle lenses with aspherical lenslets. British Journal of Ophthalmology. 2021:bjophthalmol-2020-318367. doi: 10.1136/bjophthalmol-2020-318367 33811039 PMC9340037

[24] Yam JC, Jiang Y, Tang SM, Law AKP, Chan JJ, Wong E, et al. Low-Concentration Atropine for Myopia Progression (LAMP) Study: A Randomized, Double-Blinded, Placebo-Controlled Trial of 0.05%, 0.025%, and 0.01% Atropine Eye Drops in Myopia Control. Ophthalmology. 2019;126(1):113–24. doi: 10.1016/j.ophtha.2018.05.029 . 30514630

[25] Li FF, Zhang Y, Zhang X, Kei Yip BH, Tang SM, Kam KW, et al. Age effect on treatment responses to 0.05%, 0.025%, and 0.01% atropine: Low-concentration Atropine for Myopia Progression (LAMP) Study. Ophthalmology. 2021. Epub 2021/01/11. doi: 10.1016/j.ophtha.2020.12.036 . 33422558

[26] Joachimsen L, Farassat N, Bleul T, Böhringer D, Lagrèze WA, Reich M. Side effects of topical atropine 0.05% compared to 0.01% for myopia control in German school children: a pilot study. Int Ophthalmol. 2021;41(6):2001–8. Epub 2021/02/27. doi: 10.1007/s10792-021-01755-8 ; PubMed Central PMCID: PMC8172502. 33634343 PMC8172502

[27] Rawlins M. De testimonio: on the evidence for decisions about the use of therapeutic interventions. Lancet. 2008;372(9656):2152–61. doi: 10.1016/S0140-6736(08)61930-3 19101391

[28] Myles W, Dunlop C, McFadden SA. The Effect of Long-Term Low-Dose Atropine on Refractive Progression in Myopic Australian School Children. J Clin Med. 2021;10(7). Epub 2021/05/01. doi: 10.3390/jcm10071444 ; PubMed Central PMCID: PMC8036859. 33916204 PMC8036859

[29] Chia A, Chua WH, Wen L, Fong A, Goon YY, Tan D. Atropine for the Treatment of Childhood Myopia: Changes after Stopping Atropine 0.01%, 0.1% and 0.5%. Am J Ophthalmol. 2013. doi: 10.1016/j.ajo.2013.09.020 24315293

[30] Polling JR, Tan E, Driessen S, Loudon SE, Wong HL, van der Schans A, et al. A 3-year follow-up study of atropine treatment for progressive myopia in Europeans. Eye (Lond). 2020;34(11):2020–8. Epub 2020/09/23. doi: 10.1038/s41433-020-1122-7 ; PubMed Central PMCID: PMC7785025. 32958872 PMC7785025

[31] Ruiz-Pomeda A, Prieto-Garrido FL, Hernandez Verdejo JL, Villa-Collar C. Rebound Effect in the Misight Assessment Study Spain (Mass). Curr Eye Res. 2021. Epub 2021/01/19. doi: 10.1080/02713683.2021.1878227 . 33460537

